# In Silico Identification and In Vitro Validation of Repurposed Compounds Targeting the RSV Polymerase

**DOI:** 10.3390/microorganisms11061608

**Published:** 2023-06-18

**Authors:** Eric Xu, Seohyun Park, Juan Calderon, Dongdong Cao, Bo Liang

**Affiliations:** Department of Biochemistry, Emory University School of Medicine, Atlanta, GA 30322, USA

**Keywords:** Respiratory Syncytial Virus (RSV), RNA-dependent RNA Polymerase (RdRP), micafungin, totrombopag, verubecestat, in silico simulation, in vitro transcription assay

## Abstract

Respiratory Syncytial Virus (RSV) is the top cause of infant hospitalization globally, with no effective treatments available. Researchers have sought small molecules to target the RNA-dependent RNA Polymerase (RdRP) of RSV, which is essential for replication and transcription. Based on the cryo-EM structure of the RSV polymerase, in silico computational analysis including molecular docking and the protein-ligand simulation of a database, including 6554 molecules, is currently undergoing phases 1–4 of clinical trials and has resulted in the top ten repurposed compound candidates against the RSV polymerase, including Micafungin, Totrombopag, and Verubecestat. We performed the same procedure to evaluate 18 small molecules from previous studies and chose the top four compounds for comparison. Among the top identified repurposed compounds, Micafungin, an antifungal medication, showed significant inhibition and binding affinity improvements over current inhibitors such as ALS-8112 and Ribavirin. We also validated Micafungin’s inhibition of the RSV RdRP using an in vitro transcription assay. These findings contribute to RSV drug development and hold promise for broad-spectrum antivirals targeting the non-segmented negative-sense (NNS) RNA viral polymerases, including those of rabies (RABV) and Ebola (EBOV).

## 1. Introduction

The Respiratory Syncytial Virus (RSV) is the most prominent cause of infant hospitalization, bronchiolitis, and pneumonia in the United States, while also being a major cause of hospitalization for immunocompromised individuals and the elderly [1]. RSV is a member of the *Pneumoviridae* family in the order *Mononegavirales*, which has a negative-sense RNA genome encapsidated by nucleoproteins [2]. The virus is highly contagious through the respiratory route, leading down to the nasopharynx and upper respiratory tract.

The L protein, the catalytic core of the RNA-dependent RNA Polymerase (RdRP), is usually a primary target against viral infection. The RdRP begins replication via cis-acting sequences in the Le region adjacent to the 3′ terminus [3]. In transcription, the polymerase starts at the 3′ terminus and stops at the gene end before restarting at gene start signals, synthesizing messenger RNA (mRNA) along the nucleocapsid [3]. During replication, the polymerase ignores the gene junctions and synthesizes a full-length complementary antigenome RNA; the encapsidated antigenome then increases polymerase processivity to allow RNA synthesis. Research on RdRP inhibition is limited, with only one structural class of inhibitors reaching clinical testing [4]. Currently, the only medication that has been FDA-approved for RSV polymerase is ribavirin [2]. Unfortunately, ribavirin is expensive and controversial due to the mechanism of its antiviral activity, causing the drug to be abandoned due to limited efficacy and strong adverse effects. 

Therefore, a more efficient screening assay is necessary to identify additional RdRP inhibitors for the faster therapeutic and prophylactic development of potential drug-like inhibitors. Computational methods and advancements in technology, such as cryo-electron microscopy and computer-aided drug discovery (CADD), can be harnessed for faster target identification and validation, as well as hit-to-lead molecule generation, reducing the risk, time, and cost associated with in vitro testing [5]. In recent years, in silico studies have played a key role in drug development, particularly in the discovery of potential therapeutics for various viruses. These computational approaches have enabled researchers to better understand viral structures, functions, and interactions with host cells, facilitating the identification of potential drug targets and the design of effective antiviral agents. 

For example, in silico procedures have played a critical role in identifying the potential drug candidates against the Hepatitis C virus (HCV). Previous studies have employed computational methods, such as molecular docking and virtual screening, to identify novel inhibitors of the HCV NS3/4A protease: an essential enzyme for viral replication. Several compounds with a high binding affinity and promising inhibitory activity have been identified that are comparable with currently approved HCV drugs such as simeprevir and grazoprevir [6]. In silico techniques have also contributed significantly to the development of drugs targeting the Human Immunodeficiency virus-1 (HIV-1) integrase enzyme, which is crucial for viral replication. Computational approaches, including homology modeling, molecular docking, and virtual screening, have allowed researchers to investigate the enzyme’s structure and function, leading to the identification of potential integrase inhibitors [7]. 

These examples demonstrate the significant contributions of in silico studies in antiviral drug development for various viruses, providing strong support for the use of computational approaches in the search for effective therapeutics against RSV. By leveraging the power of in silico experimentation, the drug discovery process has been accelerated to help quickly identify novel drug candidates and develop more effective treatments for RSV and other viral infections.

In this study, we chose the ChEMBL database (https://www.ebi.ac.uk/chembl/, accessed on 4 January 2022) of 2.4 million compounds and selected 6554 inhibitor candidates that had undergone clinical trials from Phases 1 to 4, ensuring minimal cytotoxicity. After reducing these to 4919 candidates by an automatic elimination of flexible ligands, we calculated and ranked the compounds’ binding affinities, extracting the top ten candidates. We also evaluated 18 previously studied compounds using a similar computational analysis and then selected four as a comparison group. Micafungin, Totrombopag, and Verubecestat emerged as the most promising repurposed compound candidates based on their binding affinities. These three compounds had never been tested against RSV polymerase, demonstrating the effectiveness of utilizing repurposed compounds in silico for drug discovery. We then validated some of those top repurposed compound candidates using an in vitro assay, in which Micafungin exhibited significant inhibitory activity, while Verubecestat showed minor inhibition. Studying these repurposed compounds allowed us to identify new leads targeting the RSV polymerase and held promise for broad-spectrum antivirals targeting the polymerases of non-segmented negative-sense (NNS) viruses such as rabies (RABV) and Ebola (EBOV).

## 2. Materials and Methods

### 2.1. Preparation of the RSV Polymerase Structure

The three-dimensional cryo-EM structure of RSV polymerase strain A2 was obtained from the Protein Data Bank (PDB) with PDB ID 6UEN [8]. The structure was processed using the Molecular Graphics Library tools (MGLTools) within AutoDock 4.2 [9]. Polar hydrogen atoms and Kollman charges were added to the protein, and water molecules were removed from the protein structure to clear possible binding pockets. The structure was then checked for missing residues and optimized to produce a suitable structure for docking. The prepared RSV polymerase structure was then saved as a PDBQT file. 

### 2.2. Small Molecule Procurement

The ChEMBL library, containing around 2.5 million compounds, was filtered to 6554 molecules which are in phases one to four of clinical trials to minimize cytotoxicity. Open Babel [10] on Linux was utilized to split the downloaded SDF file into individual SDF files for every molecule. Each individual SDF file was then optimized in Open Babel using Obminimize to perform geometry optimization and minimize each small molecule’s potential energy. We used the Merck (Rahway, NJ, USA) Molecular Force Field 1994 (MMFF94) with a 1000 maximum number of steps in the energy minimization algorithm for good accuracy on small molecules. Each individual SDF file was then converted into a PDBQT file format for AutoDock compatibility.

### 2.3. Binding Affinity Measurements

Prepared ligands were docked into the active pocket of RSV polymerase by creating a grid box around the entire RSV polymerase structure using AutoGrid. The grid size was chosen as 80 × 80 × 80 Å. A configuration file was created with exhaustiveness = 8 and energy range = 10 for accurate docking without sacrificing the computational time. AutoDock Vina [11] was then implemented on Linux to determine the top 10 binding poses for each small molecule on the protein, and its corresponding binding affinity was measured in kcal/mol. Due to the inability of the program to compute multiple molecules sequentially, each molecule was automated and docked using a BATCH file to execute a Perl script, which cycled through each individual small molecule PDBQT file to perform protein-ligand docking.

### 2.4. Inhibition Measurements

We utilized the MGLTools within AutoDock 4.2 to calculate the inhibition constants of the top ten repurposed compounds. The same PDBQT file of RSV RdRP was used for individual protein-ligand docking. Gasteiger charges were added to each repurposed compound. The same grid coordinates and dimensions from automated docking were used. In total, 50 Genetic Algorithm runs with a population size of 300 were configured so that the program would return 50 inhibition constant (Ki) trials measured in nanomoles. The Lamarckian Genetic Algorithm was then selected to run docking for each individual repurposed compound. 

### 2.5. Hydrogen Bond Analysis

The resulting PDBQT files from AutoDock 4.2 were transformed into PDB files using Open Babel. The PDB files were uploaded to the Protein–Ligand Interaction Profiler from the Dresden University of Technology [12]. Once uploaded, the program output hydrogen bonds and notable interactions between the ligand and the RSV polymerase.

### 2.6. In Vitro RNA Synthesis Assay Screening Inhibitors

The 12-nt trailer complementary sequence (TrC12) at the 3′ terminal of the antigenome was used as a template in the RNA synthesis assay. All RNA oligonucleotides were chemically synthesized by Integrated DNA Technologies (Coralville, IA, USA). Radioactive isotope-labeled nucleotides [α-^32^P] GTP and [γ-^32^P] ATP were purchased from Perkin Elmer (Waltham, MA, USA). Small molecules were purchased from MedChemExpress (Monmouth Junction, NJ, USA). The reaction mixtures contained a 2 μM RNA template TrC12, the RSV L-P complexes (∼300 ng), NTPs (ATP, CTP, and UTP each at 1.25 mM and GTP at 50 μM with 5 μCi of [α-^32^P]GTP), 1 mM small molecule in 10% DMSO (10% DMSO solution was used as a negative control), and a reaction buffer (50 mM Tris-HCl pH 7.4, 8 mM MgCl_2_, 5 mM dithiothreitol, 10% glycerol) at a final volume of 20 μL. The reaction mixtures were incubated at 30 °C for 2 h and heated to 90 °C for 3 min, and then 5 μL of the stop buffer (90% formamide, 20 mM EDTA, 0.02% bromophenol blue) was added to each reaction mixture. The RNA products were analyzed by electrophoresis on a 20% polyacrylamide gel containing 7 M urea in a Tris-borate-EDTA buffer, followed by phosphorimaging with a Typhoon FLA 7000 scanner (GE Healthcare, Chicago, IL, USA). The quantification of these images was carried out with an analysis toolbox from ImageQuant TL 7.0 software (GE Healthcare, Chicago, IL, USA). We analyzed the images using area- and profile-based tools and selected the corresponding area of each lane with a box for calculation by the software. The molecular weight ladders were generated by labeling Tr7, Tr14, Tr21, and Tr25 with [γ-^32^P] ATP using polynucleotide kinase and following the protocols according to the manufacturer (NEB).

## 3. Results

### 3.1. In Silico Candidate Extraction Procedure

**Figure 1:**
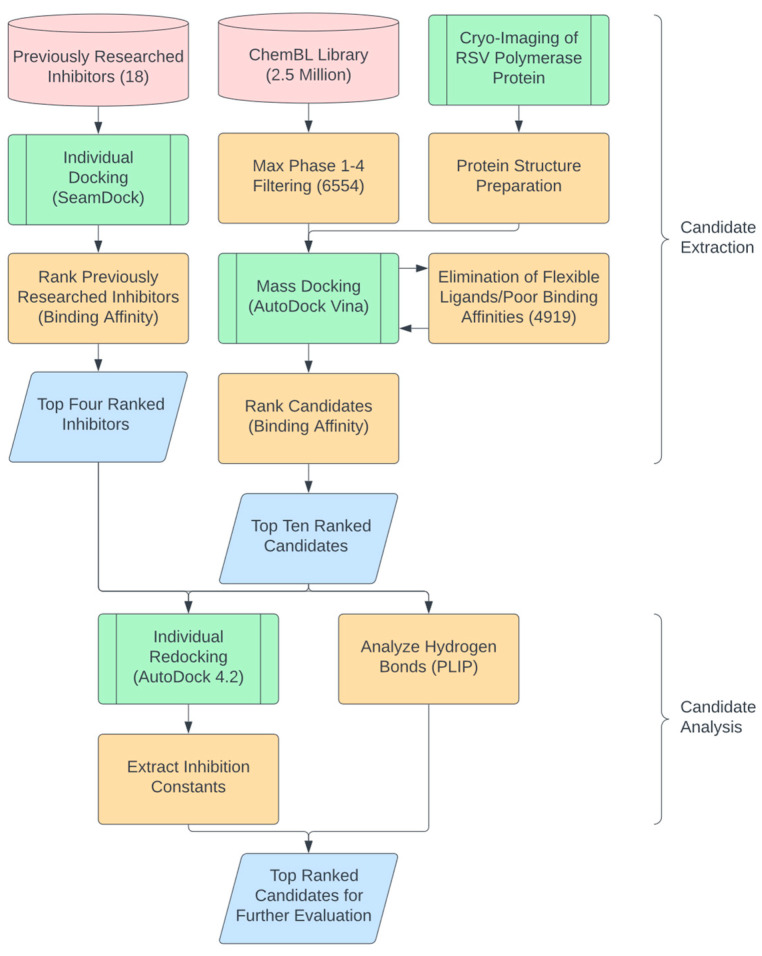
Flowchart for in silico candidate extraction, validation, and analysis. Cylinders represent data sources (i.e., The ChEMBL Library, https://www.ebi.ac.uk/chembl/, accessed on 4 January 2022), parallelograms represent specific data points, green nodes represent major processing steps, and orange nodes represent intermediatory steps. The output represents the top candidates of the selection process that were on-par or more inhibitory than the previously researched inhibitors.

The ChEMBL library (2,354,965 compounds) was filtered to 6554 compounds to select candidates that had proceeded through any phase 1–4 clinical trials to avoid inhibitors with potential cytotoxicity (Figure 1). The cryo-EM structure of the RSV strain A2 polymerase (PDB: 6UEN) was obtained from the PDB database and then prepared for AutoDock Vina by removing water molecules and adding Kollman charges. Mass docking by AutoDock Vina was then performed on the RdRP, resulting in the relative binding affinity values (in kcal/mol) for each compound. Compounds with flexible ligand structures or poor binding affinities were automatically eliminated by AutoDock Vina, narrowing the index to 4919 candidates (Figure 1). The new candidates were then ranked based on their binding affinities, with a lower binding affinity exhibiting higher efficacy. As a comparison group, 18 previously tested inhibitors for RdRP [3,13,14,15,16,17,18,19,20,21] were also ranked (according to their binding affinities) via individual docking through SeamDock (Figure 1). Four of these inhibitors were selected and used as a basis for evaluating the effectiveness of new candidates. We pooled the top ten repurposed compound candidates and the four previously studied compounds before performing individual redocking on the compounds using AutoDock 4.2, extracting precise binding affinities and inhibition constants (Figure 1). The Proteins Ligand Interaction Profiler (PLIP) was then employed to analyze the number of hydrogen bonds within the structures of the best poses of the compounds. An increase in the number of hydrogen bonds between the small molecule and the protein resulted in higher oral bioavailability, which reduced the amount of the administered small molecule for a desired pharmacological response and could reduce the risk of side effects and toxicity. This property also allowed for drug absorption and efficient systemic circulation [22].

### 3.2. In Silico Candidate Results

**Figure 2:**
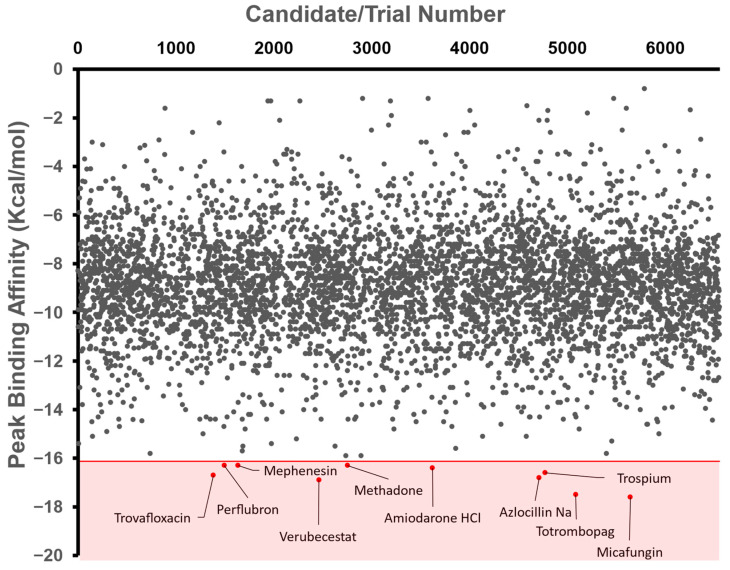
Mass Docking and Selection Criteria. Free binding affinity (kcal/mol) was calculated using AutoDock Vina and then automated via a BATCH file. Tested compounds came from the ChEMBL Library (2,354,965) and then were filtered to molecules currently proceeding through clinical trials (Max Phase 1–4), resulting in an input database of 6554 compounds. These were further narrowed to around 4919 compounds due to the automatic elimination of flexible ligands and positive binding affinities by AutoDock Vina. The shaded region represents the region of our selection criteria for the 10 candidates with the lowest binding affinities, with labels beside the corresponding compounds. The number above represents the arbitrary trial number, which was then used to identify the compound.

The top ten repurposed compound candidates with the lowest free affinities were Micafungin, Totrombopag, Verubecestat, Trovafloxacin, Azlocillin Na (Azlocillin Sodium), Trospium, Amiodarone HCl (Amiodarone Hydrochloride), Perflubron, Mephenesin, and Methadone (Figure 2). Each of these compounds are repurposed drug: Micafungin is an antifungal agent that is used to treat a broad range of fungi strains [23]. Totrombopag is a thrombopoietin receptor agonist that stimulates platelet production in patients with chronic immune thrombocytopenia and aplastic anemia [24]. Trovafloxacin is a fluoroquinolone antibiotic that is used to treat various bacterial infections [25]. Azlocillin Na is a broad-spectrum penicillin antibiotic that is effective against a wide range of bacteria, including those causing respiratory and urinary tract infections [26]. Trospium is an anticholinergic medication used to treat overactive bladder symptoms [27]. Amiodarone HCl is an antiarrhythmic medication that is used to treat and prevent certain types of abnormal heart rhythms [28]. Perflubron is a fluorocarbon compound used as a contrast agent in diagnostic imaging procedures, particularly for liver imaging [29]. Mephenesin is a muscle relaxant used to alleviate muscle spasms and stiffness associated with certain musculoskeletal conditions [30]. Methadone is a synthetic opioid medication that is primarily used for the treatment of opioid dependence and chronic pain management [31].

The top four inhibitors found from previous research were PC786, AZ-27, Ribavirin, and ALS-8112. PC786 is a nonnucleoside inhibitor targeting the RSV L protein by inhalation, which is currently undergoing Phase 2 of clinical trials [20]. Similarly, AZ-27 is also a nonnucleoside inhibitor of the L protein and has demonstrated strong antiviral capabilities against both A and B strains of RSV [32]. ALS-8112 shows promise in preclinical studies and is the parent molecule of ALS-8176: another inhibitor of RSV polymerase [33]. Ribavirin is a current broad-spectrum antiviral medication that is used to treat several viral infections, including RSV and is currently approved for the treatment of RSV infections in certain patient populations [34].

**Figure 3:**
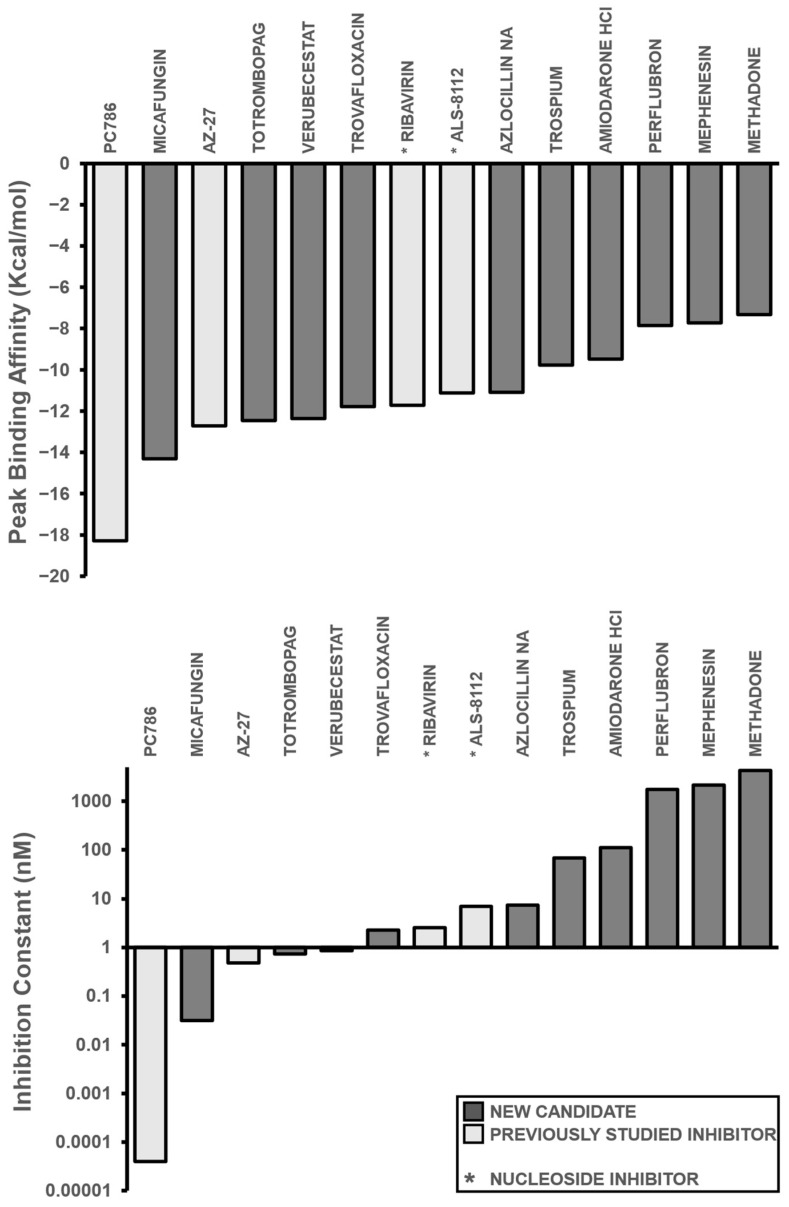
Binding Affinity and Inhibition Constant of Compounds. (**a**) Binding affinities (kcal/mol) were first calculated via AutoDock Vina for relative measurement before individual redocking with AutoDock 4.2 for validation. The inhibitors are displayed in ascending order, with the previously studied inhibitors referenced in white and new candidates in black. (**b**) Individual inhibition constants (K_i_, measured in nM) of the selected inhibitors were calculated via AutoDock 4.2 and then plotted in ascending order within a logarithmic scale. A lower inhibition constant relates to higher inhibition efficiency. Bars that are shaded in dark grey represent candidate leads, while bars shaded in light grey represent inhibitors from the literature. An asterisk before the name of each small molecule denotes that the small molecule is a nucleoside inhibitor.

After calculating the precise binding affinities (Figure 3a) and inhibition constants of the aforementioned small molecules (Figure 3b), the inhibitors were then placed in ascending order based on their inhibition constants (in nM): PC786 (0.0000399), Micafungin (0.0317), AZ-27 (0.47997), Totrombopag (0.73929), Verubecestat (0.85487), Trovafloxacin (2.27), Ribavirin (2.56), ALS-8112 (6.98), Azlocillin Na (7.47), Trospium (68.41), Amiodarone HCl (111.15), Perflubron (1760), Mephenesin (2160), and Methadone (4300) (Table 1).

Among the ten candidates, three were comparable or showed more inhibition than the comparison group: Micafungin, Totrombopag, and Verubecestat. Micafungin demonstrated a significant improvement to both the free binding affinity and inhibition constant over several current inhibitors; however, PC786 still had more efficacy. Micafungin had a binding affinity of −14.32 kcal/mol and an inhibition constant of 0.0317 nM, which showed considerable improvement over inhibitors such as AZ-27, ALS-8112, and Ribavirin, with binding affinities of −12.71, −11.72, and −11.13 kcal/mol, and inhibition constants of 0.47997, 2.56, and 6.98 nM, respectively (Table 1). By contrast, Verubecestat and Totrombopag showed comparable results to current inhibitors with binding affinities of −12.37 and −12.46 kcal/mol and inhibition constants of 0.85 nM and 0.73929 nM (Table 1). These compounds are undergoing clinical trials and have been administered to volunteers, so their delivery should not pose a risk in terms of cytotoxicity; however, in vitro testing is required to truly test the pharmacokinetic response of these compounds as well as to validate the specific inhibition values of each top repurposed compound candidate lead.

### 3.3. Potential Molecular Actions of Top Repurposed Compound Candidates

Micafungin is an approved broad-spectrum antifungal medication that is used for the treatment of invasive candidiasis, esophageal candidiasis, and the prophylaxis of Candida infections in patients undergoing hematopoietic stem cell transplantation. Micafungin belongs to a class of antifungal drugs called echinocandins, which inhibit the synthesis of β (1,3)-d-glucan, a component of the fungal cell wall, leading to cell death. The chemical formula of micafungin is C56H71N9O23S, and its molecular weight is 1270.29 g/mol [35].

Totrombopag is currently in phase 2 clinical trials and is a medication used to treat a low platelet count in patients with chronic immune thrombocytopenia (ITP) and chronic hepatitis C-associated thrombocytopenia. Totrombopag is a thrombopoietin receptor agonist that stimulates the production and maturation of platelets from bone marrow megakaryocytes [24]. The chemical formula of Totrombopag is C25H22N8O2, and its molecular weight is 466.51 g/mol.

Verubecestat is a phase 3 investigational medication that belongs to a class of drugs called beta-secretase inhibitors (BSIs). It is being developed for the treatment of Alzheimer’s disease. Verubecestat is a small molecule inhibitor that selectively targets the beta-site amyloid precursor protein cleaving enzyme 1 (BACE1): an enzyme responsible for the cleavage of the amyloid precursor protein (APP) into beta-amyloid (Aβ) peptides [36]. Its chemical formula is C17H17F2N5O3S, and its molecular weight is 409.42 g/mol.

### 3.4. In Vitro Assay Validation

**Figure 4:**
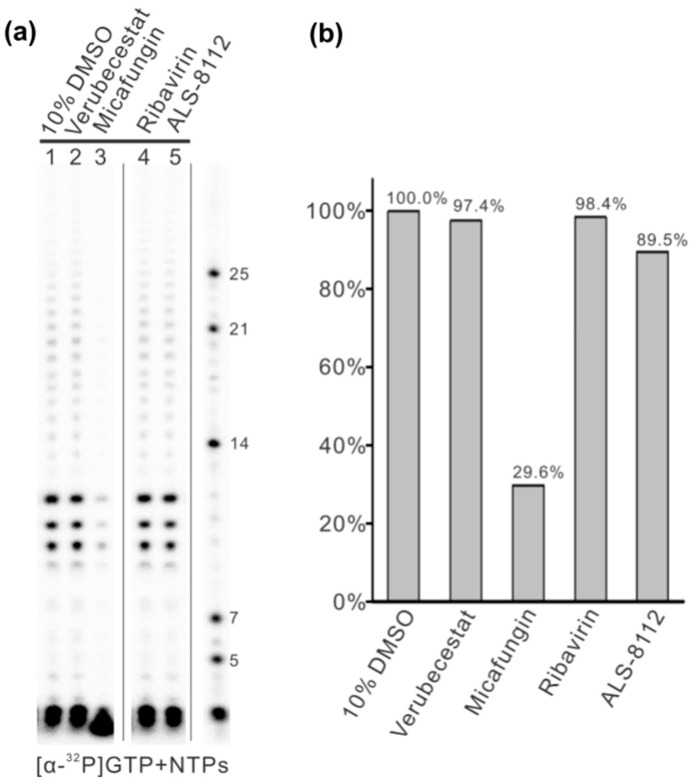
The in vitro RNA synthesis assay by RSV polymerase testing with different small molecules using 12-nt trailer complementary (TrC12) as the template. (**a**) Verubecestat, Micafungin, Filibuvir, Ribavirin, ALS-8112, and ALS-8176 at a concentration of 1 mM was tested with RSV polymerase using TrC12 as a template in the presence of NTPs (ATP, CTP, and UTP each at 1.25 mM and GTP at 50 μM with 5 μCi of [α-32P] GTP). All the small molecules were dissolved in 10% dimethyl sulfoxide (DMSO), and 10% DMSO was used as a negative control. The right lane shows the molecular weight ladder. (**b**) Total polymerase activities from panel A were quantified and plotted. The quantification of the images was carried out with an analysis toolbox from ImageQuant TL 7.0 software (GE Healthcare). We analyzed the images using area- and profile-based tools and selected the corresponding area of each lane with a box for calculation by the software.

Based on our in silico experiment results, we selected some small molecules as inhibitor candidates, such as Micafungin, Verubecestat, Ribavirin, and ALS-8112, and applied them to an in vitro RNA synthesis assay (Figure 4). These small molecules were dissolved in 10% dimethyl sulfoxide, and a 10% dimethyl sulfoxide solution was used as a negative control. The 12-nt trailer complementary sequence (TrC12, 3′-UGCUCUUUUUUU) at the 3’ terminal of the antigenome was used as a template in the RNA synthesis assay. The control sample showed enriched bands of 10–12 nts and some weak bands larger than 12 nts, which were polyadenylated RNA products (Figure 4, lane 1). The impacts of Verubecestat, Ribavirin, and ALS-8112 on the RNA synthesis activity of RSV polymerase were not significant, with 97.4%, 98.4%, and 89.5% of the control group activity (Figure 4, lanes 2, 4, and 5). However, Micafungin, the best candidate among the selected small molecules, showed significant inhibition activity toward RSV polymerase, which retained only 29.6% of the activity of the control group (Figure 4, lane 3).

## 4. Discussions

Respiratory syncytial virus (RSV) continues to pose a significant threat to global health, particularly in infants, the elderly, and immunocompromised individuals. Despite its high prevalence and associated morbidity, no specific antiviral drugs or universally effective vaccines have yet been approved for RSV, highlighting an urgent need for faster drug discovery strategies. In this context, our study explores the use of computational methods to identify potential inhibitors of the RSV polymerase: a key enzyme in the virus’s replication process. 

Different computational methods were utilized in this study, such as molecular docking software, to efficiently screen and identify the potential inhibitors of the RSV polymerase. The primary advantage of initially using a computational approach is its capacity and ability to rapidly and methodically screen a relatively larger database containing a variety of chemical compounds and small molecules to output a theoretical binding affinity to the target protein of interest. Through this swift approach, researchers can quickly identify potential candidate compounds out of a sizable dataset, which may be impractical to achieve using traditional laboratory-based methods due to the high costs and time requirements related to in vitro target validation [11]. 

AutoDock Vina and AutoDock 4.2 leverage robust scoring functions, and optimization algorithms were used to effectively simulate molecular interactions and predict binding affinities between potential drugs and target proteins. The tools’ optimization algorithms, including the Broyden-Fletcher-Goldfarb-Shanno Method employed by AutoDock Vina and the Lamarckian Genetic Algorithm used by AutoDock 4.2, adeptly explored the conformational space of protein–ligand complexes [37]. However, computational methods come with their own set of challenges, and solely relying on the outputs of such methods to create conclusions can generate inaccurate results. While AutoDock bases its binding affinity estimations on genomic and chemical data, much of the calculations are conducted from basic assumptions and simplifications. 

Furthermore, there may also be sources of error attributed to the computational simulation, such as an inappropriate docking box. Blind docking was attempted when creating the parameters for the grid box during large-library automated docking, but when docking via AutoDock 4.2, the grid box was not able to cover the entire surface of the protein, resulting in protruding residues. This was problematic because if the size and location of the grid box were not positioned over a potential binding site, then the ligand could not find a binding pose on the residues that were not covered. To combat these limitations concerning the docking parameters within the configuration file, we set the energy range in the configuration file to ten, allowing AutoDock Vina to generate ten different binding affinities of each small molecule, and we then employed an exhaustiveness of eight for a good balance between accuracy and computation time [38]. Moreover, among the 1635 small molecules that were not docked, some of the small molecules could also be flexible ligands, which is a limitation for AutoDock Vina. Flexible ligand docking can be challenging due to the degree of computational complexity, accuracy, and difficulties of automating the process when working with a large library of small molecules, despite there being alternative approaches. Therefore, it is critical to couple efficient in silico results with in vitro assays to obtain accurate evidence because of the limitations that may arise when utilizing computational methods. 

Regarding our study, we created a reproducible drug discovery pathway to find the potential inhibitors of RSV RdRP (which could be used for other viral polymerases) with the use of both in silico and in vitro methodologies. During the computational process, we were able to identify one small molecule that exhibited exemplary inhibitory properties: Micafungin. Through our computational process, Micafungin was shown to have an inhibition constant of 0.0317 nM, and the in vitro RNA synthesis assay further supported the inhibitory effects of Micafungin, as the RSV polymerase affected by Micafungin only retained 29.6% of the activity of the control group. Furthermore, the inhibition efficacy of Micafungin against RSV polymerase had not been experimentally evaluated before, representing a significant gap in the current understanding of the therapeutic applications of this compound. 

Micafungin has had precedent cases that have demonstrated antiviral activities, such as its potential to inhibit Enterovirus 71 infection; this compound was able to reportedly target virus replication with an estimated IC_50_ of 5–8 µM to an EV71 replicon in Vero cells [39]. Other studies have also shown that Micafungin inhibits Zika virus [40] and Dengue virus serotype 2 [41] with an IC50 of 7.35 and 10.23 µM, respectively. In addition, studies have started to tie Micafungin with respiratory antiviral inhibition. A recent study tested the antiviral effects of Micafungin against the SARS-CoV-2 Wuhan strain with an IC_50_ of 26.1 µM [42]. Therefore, we believe that Micafungin shows a strong indication to be a potential broad-spectrum antiviral due to precedent cases involving antiviral activities toward other respiratory viruses and with this study. Previous studies have shown that the RNA synthesis catalytic domains of the L proteins of NNS viruses share high similarities [43,44]; therefore, we speculated that Micafungin could have significant inhibitory effects against other NNS viruses such as rabies and Ebola, similar to RSV.

Although our in silico procedures also identified Verubecestat as a possible inhibitor of RSV polymerase, our in vitro assay validation did not support the theoretical antiviral activity of this compound because the RSV polymerase retained 97.4% of the control group activity. Moreover, a drug delivery pathway needs to be defined for the repurposed Micafungin or Verubecestat compounds to effectively inhibit RSV, and additional ADMET assays are needed to predict the cytotoxicity in vivo. Further experimentation should be performed using a much larger data set by including small preclinical molecules from the ChEMBL database to allow for the discovery of even more candidates. Furthermore, the use of the Schrodinger Drug Discovery suite could be used to work around flexible ligand docking and molecular dynamics simulations could give key information about the protein’s flexibility and protein–ligand interactions, such as how the small molecule binds to the target protein and how the small molecule induces conformational changes to the protein. This step could facilitate the modification and optimization of drug candidates to improve their binding affinities.

## 5. Patents

A U.S. provisional patent application is in preparation.

## Figures and Tables

**Table 1:** Bond Numbers and Chemical Structures of Selected Inhibitors. Binding Affinities (kcal/mol) and Inhibition Constants (Ki, in nM) were measured in AutoDock 4.2, while the numbers of hydrogen bonds were recorded with a Protein–Ligand Interaction Profiler.

Name	Chemical Structure	Inhibition Constant (nM)	Peak Binding Affinity (kcal/mol)	Number of Hydrogen Bonds	Nucleoside/ Non-Nucleoside
PC786 *	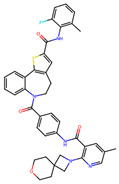	39.9 × 10^−6^	−18.28	6	Non-Nucleoside
MICAFUNGIN	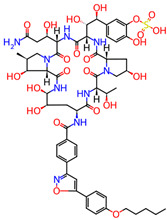	0.0317	−14.32	0	Non-Nucleoside
AZ-27 *	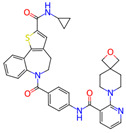	0.47997	−12.71	5	Non-Nucleoside
TOTROMBOPAG	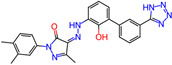	0.73929	−12.46	6	Non-Nucleoside
VERUBECESTAT	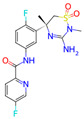	0.85487	−12.37	5	Non-Nucleoside
TROVAFLOXACIN	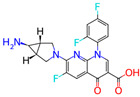	2.27	−11.79	7	Non-Nucleoside
RIBAVIRIN *	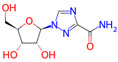	2.56	−11.72	6	Nucleoside
ALS-8112 *	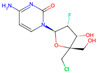	6.98	−11.13	1	Nucleoside
AZLOCILLIN NA	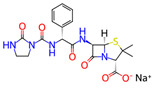	7.47	−11.09	1	Non-Nucleoside
TROSPIUM	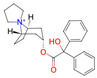	68.41	−9.77	5	Non-Nucleoside
AMIODARONE HCL	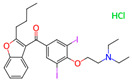	111.15	−9.49	5	Non-Nucleoside
PERFLUBRON	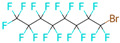	1760	−7.85	1	Non-Nucleoside
MEPHENESIN	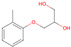	2160	−7.73	3	Non-Nucleoside
METHADONE	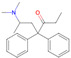	4300	−7.32	9	Non-Nucleoside

* These compounds are previously researched RSV inhibitors.

## Data Availability

The data supporting the findings of this study are available from the corresponding author B.L. on request.
